# Anti-angiogenic drug scheduling optimisation with application to colorectal cancer

**DOI:** 10.1038/s41598-018-29318-5

**Published:** 2018-07-25

**Authors:** M. Sturrock, I. S. Miller, G. Kang, N. Hannis Arba’ie, A. C. O’Farrell, A. Barat, G. Marston, P. L. Coletta, A. T. Byrne, J. H. Prehn

**Affiliations:** 1 Department of Physiology and Medical Physics, Centre for Systems Medicine, Royal College of Surgeons in Ireland, 123 St Stephens Green, Dublin 2, Ireland; 2 School of Medicine, University of Leeds Brenner Building, St James’s University Hospital, Leeds, LS9 7TF UK; 3 Conway Institute, University College Dublin, Belfield, Dublin 4, Ireland; 4 Liverpool Hope University, Hope Park, Liverpool, L16 9JD UK

## Abstract

Bevacizumab (bvz) is a first choice anti-angiogenic drug in oncology and is primarily administered in combination with chemotherapy. It has been hypothesized that anti-angiogenic drugs enhance efficacy of cytotoxic drugs by “normalizing” abnormal tumor vessels and improving drug penetration. Nevertheless, the clinical relevance of this phenomenon is still unclear with several studies over recent years suggesting an opposing relationship. Herein, we sought to develop a new computational tool to interrogate anti-angiogenic drug scheduling with particular application in the setting of colorectal cancer (CRC). Specifically, we have employed a mathematical model of vascular tumour growth which interrogates the impact of anti-angiogenic treatment and chemotherapeutic treatment on tumour volume. Model predictions were validated using CRC xenografts which underwent treatment with a clinically relevant combinatorial anti-angiogenic regimen. Bayesian model selection revealed the most appropriate term for capturing the effect of treatments on the tumour size, and provided insights into a switch-like dependence of FOLFOX delivery on the tumour vasculature. Our experimental data and mathematical model suggest that delivering chemotherapy prior to bvz may be optimal in the colorectal cancer setting.

## Introduction

Solid tumours progress through two separable phases: avascular and vascular. In the avascular phase of tumour growth, nutrients and oxygen are delivered to tumour cells via diffusion processes alone from surrounding host capillaries. While in this phase, which can last for a period of several months or even years without causing any serious damage to the host, tumour growth is limited to just a few millimetres (1–2 mm^1^) and cell proliferation is balanced by cell death^2^. Subsequently, the tumour mass may reach a critical point of transition from the avascular to vascular phase and develop an intrinsic blood supply network (angiogenesis) which supports further growth and ultimately metastases. During this process, tumour cells secrete angiogenic factors such as vascular endothelial growth factor (VEGF) in response to diminished oxygen levels and the ‘angiogenic switch’ occurs. This ‘switch’ is further influenced by biophysical triggers including metabolic and mechanical stress^2^ as well as other endogenous pro and anti-angiogenic molecules.

Bevacizumab (bvz) is an anti-VEGF humanized monoclonal antibody that targets circulating VEGF and subsequently prevents binding of VEGF to its receptors^3^ thus inhibiting angiogenesis. In most oncology settings including colorectal^4^, breast^5^ and non-small cell lung cancer^6^ bvz shows activity only when combined with cytotoxic chemotherapy. Moreover, it has been hypothesized that anti-angiogenic drugs enhance efficacy of cytotoxic drugs by “normalizing” structurally and functionally abnormal tumor vessels, thereby reducing interstitial fluid pressure and improving drug penetration^7^. Nevertheless, the clinical relevance of this phenomenon is still unclear^8^ with several studies over recent years suggesting an opposing relationship, i.e., bvz leads to a sustained decrease in the delivery of biological agents or chemotherapy, when delivered within a combinatorial regimen^9–11^. It has thus been suggested that the tumor vessel effect of anti-angiogenics (delivered in a combinatorial regimen) is likely to be time, dose and even tumour type dependent^11–13^. Further studies are therefore required to unravel these effects in a tumour and treatment specific context.

Recently, computational models have emerged as powerful tools to support the appropriate optimization of cancer therapies. Moreover, the use of mathematical models to simulate vascular tumour growth and treatments has a long history including several studies which have successfully modelled vascular tumour growth and indeed validated model predictions using experimental data sets. Two mathematical modelling studies of particular relevance^14,15^ have considered the effects of chemotherapy and anti-angiogenesis treatments on vascular tumour growth. In^15^ it is argued that administering anti-angiogenesis treatment first allows for more effective delivery of chemotherapy via pruning of ‘low flow’ vessels. In addition, using a cellular automata model, Powathil *et al*. demonstrated that the cytotoxic effect of chemotherapy is dependent on several factors such as the timing of drug delivery, the time delay between drug doses, heterogeneities of the cell cycle, spatial distribution of the tumour and the surrounding microenvironment^16,17^. Notwithstanding these studies, widespread application of cellular automaton models is difficult due to their computational cost, which further renders a comprehensive Bayesian parameter estimation study unfeasible. Significant progress has been made by incorporating a refined two-compartmental model to capture bvz pharmacokinetic properties^18^ into a previously developed vascular tumour growth model^19^. This approach was based on the ordinary differential equation model of^20^. Furthermore, the model was fitted to experimental data from four different tumour types (breast, lung, colon, head and neck). One weakness of the study (as alluded to by the authors) was that the parameter fitting was performed only locally, and different parameter sets were used to fit to the control and bvz treatment cases.

**Figure 1:**
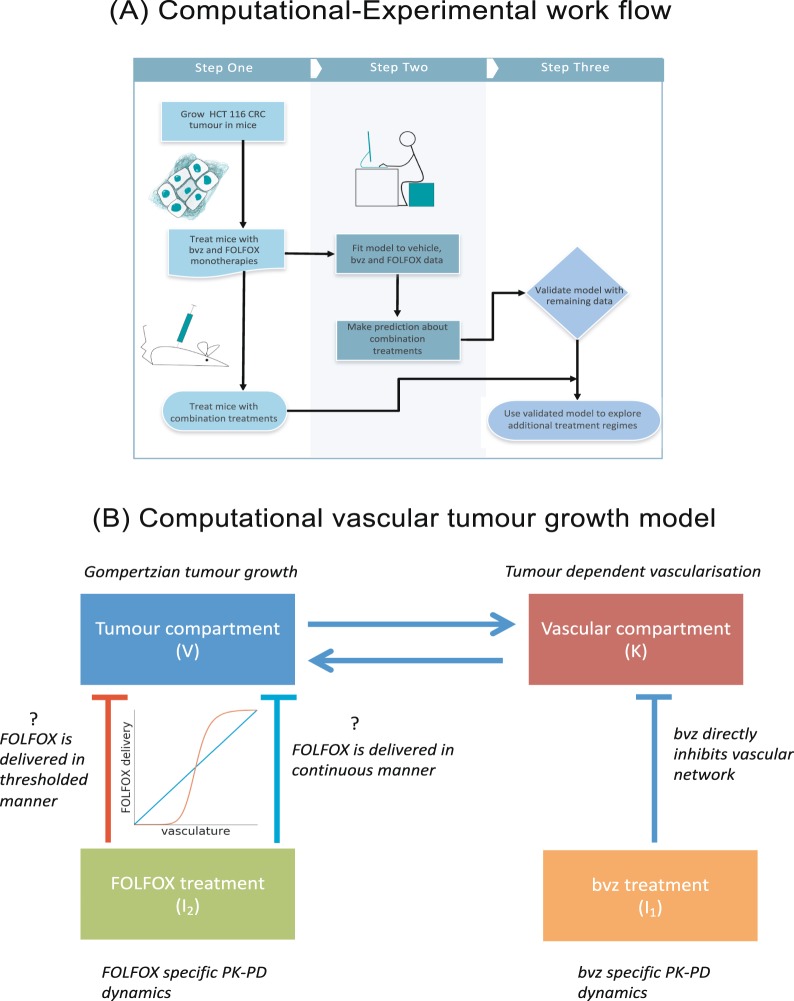
Outline of experimental work flow and tumour model. (**A**) Overview of computational-experimental work flow presented in this paper. First a CRC HCT116-luc tumour is grown in Balb/c^nu/nu^ mice before being administered with FOLFOX and bvz. Data from pre-clinical models is then used to calibrate the parameters of the computational model. These parameters are subsequently used to simulate combination treatment cases which are validated with additional experiments. This validated model is then used to explore a number of different treatment regimes. (**B**) Overview of computational vascular tumour growth model with different treatment regimes. The vascular compartment, or carrying capacity, grows in tandem with the tumour compartment. The vascular compartment also allows for the delivery of drugs. The model accounts for two different drug treatments, bvz and FOLFOX. Bvz is an anti-angiogenic drug that targets the vascular compartment and FOLFOX is a chemotherapeutic drug that targets the tumour compartment. Two different models of how FOLFOX inhibits tumour size are explored – a threshold-like dependence of delivery on the vasculature (red line in graph) and a continuous dependence of delivery on vasculature (blue line in graph). Details about model equations are explained in Methods section.

Herein, we sought to further explore anti-angiogenic drug scheduling within the setting of CRC. To address this question experimentally, we employed the gold-standard HCT116 CRC xenograft model which underwent treatment with a paradigmatic folinic acid, fluorouracil and oxaliplatin (FOLFOX) chemotherapy + anti-VEGF (bvz) regimen commonly employed in the clinical management of metastatic colorectal cancer (mCRC). With respect to the computational aspect of this work, we expanded the mathematical model of^18^ and conducted an extensive Bayesian parameter fitting of this extended model. We fitted the model to time series CRC xenograft tumour volume data obtained from vehicle treated subjects, bvz monotherapy treated subjects, and FOLFOX monotherapy treatment subjects respectively. Based on parameters found by this fitting, we made model predictions regarding the combination treatment cases which were subsequently validated in pre-clinical models. Our joint experimental-computational approach as illustrated in Fig. 1, suggests that delivery of antiangiogenic therapy after chemotherapy may deliver optimal treatment results in the setting of colorectal cancer.

## Results

### Administering FOLFOX before bvz is optimal for reducing tumour burden in the theoretical model regardless of chosen chemotherapy function form

**Figure 2:**
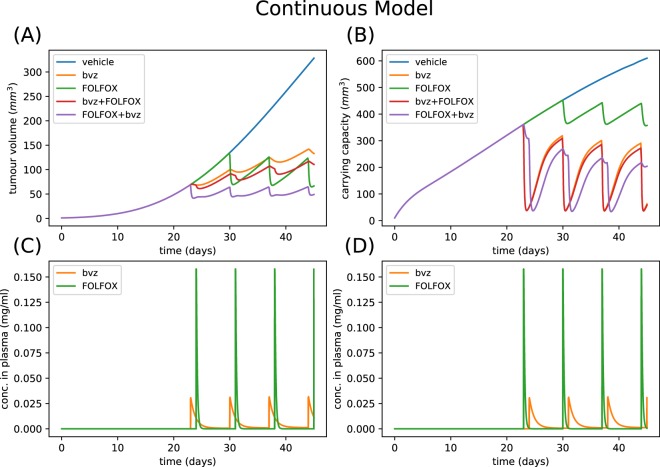
Example numerical simulation of computational vascular tumour growth model with continuous chemotherapy function. Parameters are sampled from priors displayed in Table 1. Specifically, parameters values are λ_1_ = 0.067 day^−1^, λ_2_ = 4.15 × 10^−5^ day^−1^, c = 0.04 mg/(day·mm^3p^·kg), α = 9.81 × 10^−4^ mg/(mm^3p^·kg), d = 0.47 day^−1^· mm^−2^, Bk_12_ = 0.47 day^−1^, Bk_21_ = 0.089 day^−1^, Bk_e_ = 0.79 day^−1^, Fk_12_ = 0.0014 day^−1^, Fk_21_ = 0.39 day^−1^, Fk_e_ = 0.91 day^−1^, K_F_ = 0.057 day^−1^· mm^−2^, α_1_ = 1, β_1_ = 1. (**A**) Shows how the tumour volume varies over a time period of 45 days. (**B**) Shows how the corresponding vasculature compartment or carrying capacity varies over the same time period. (**C**) Shows the bvz and FOLFOX drug concentrations in the plasma for Treatment Schedule 1 (**D**) shows the bvz and FOLFOX drug concentrations in the plasma for Treatment Schedule 2. Solutions to the ODE system are saved every 0.1 time units. The initial conditions are chosen so that the tumour volume is 1 mm^3^ and carrying capacity is 10 mm^3^ at t = 0 days.

**Figure 3:**
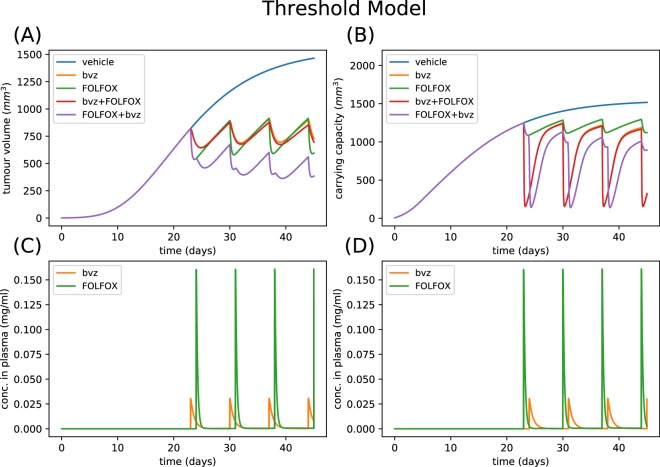
Example numerical simulation of computational vascular tumour growth model with thresholded chemotherapy function. Parameters are sampled from priors displayed in Table 1. Specifically, parameters values are λ_1_ = 0.17 day^−1^, λ_2_ = 3.39 × 10^−3^ day^−1^, c = 0.04 mg/(day·mm^3p^·kg), α = 1.46 × 10^−3^ mg/(mm^3p^·kg), d = 0.19 day^−1^·mm^−2^, Bk_12_ = 0.56 day^−1^, Bk_21_ = 0.012 day^−1^, Bk_e_ = 0.95 day^−1^, Fk_12_ = 0.22 day^−1^, Fk_21_ = 0.038 day^−1^, Fk_e_ = 0.86 day^−1^, K_F_ = 1.12 day^−1^ mm^−2^, α_2_ = 768.1 mg/nl, p_1_ = 17.95. (**A**) Shows how the tumour volume varies over a time period of 45 days. (**B**) Shows how the corresponding vasculature compartment or carrying capacity varies over the same time period. (**C**) Shows the bvz and FOLFOX drug concentrations in the plasma for Treatment Schedule 1 (**D**) shows the bvz and FOLFOX drug concentrations in the plasma for Treatment Schedule 2. Solutions to the ODE system are saved every 0.1 time units. The initial conditions are chosen so that the tumour volume is 1 mm^3^ and carrying capacity is 10 mm^3^ at t = 0 days.

It is not clear which functional form the degradation of tumour volume via FOLFOX should take, therefore, we investigated two different physiologically feasible chemotherapy function forms. Figure 2 shows the numerical simulation results of the model defined in equations 5 and 6 of the methods section using the continuous chemotherapy function (defined in equation 7) which assumes chemotherapy delivery is dependent on the vasculature in a continuous manner while Fig. 3 show the results for the threshold chemotherapy function (defined in equation 8) which assumes chemotherapy delivery is dependent on the vasculature in a switch-like manner. These simulations represent an experimentally difficult to reproduce scenario – where the tumour volumes and their vasculature are perfectly controlled between different experimental setups. The parameters were chosen so that the reduction in tumour volume caused by bvz and FOLFOX chemotherapy is similar – which helped dissect the effect of the ordering of drug delivery. Though quantitatively the two chemotherapy terms give different results, we calculated qualitative similarities in their temporal evolution. Figures 2 and 3 both show that administering bvz first resulted in a significant reduction in the carrying capacity. In terms of biology, the model solutions suggested that a reduction in vessel density may hamper the effective delivery of FOLFOX. However, the model solutions also showed that if FOLFOX is administered first, the drug is delivered effectively, due to the relative abundance of vessels, and the tumour volume decreases immediately. Hence, these numerical simulations suggested that it is optimal to deliver bvz after FOLFOX. While these mathematical model simulations are useful for carrying out thought experiments, it should not supersede careful fitting of the model to real *in vivo* data. Moreover, we were not able to choose between the two chemotherapy functions as they both gave qualitatively similar behaviour. Therefore, in the next section we present our results of fitting the mathematical model to experimental data corresponding to Treatment Schedule 1 (TS1) where bvz is given 24hrs before chemotherapy.

### The mathematical model can capture the qualitative and quantitative experimental data for monotherapies and predict the outcome of delivering bvz before FOLFOX

**Figure 4:**
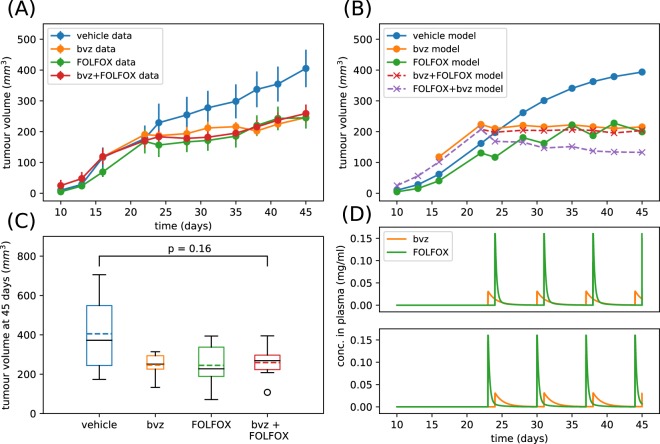
Fitting and validating the computational vascular tumour model with Treatment Schedule 1 (TS1) experimental data. Result of fitting the extended Argyri *et al*. model to experimental data from HCT116 CRC xenografts using monotherapies and TS1. Bayesian model selection was used and the threshold model was found to be the most probable given the data. Parameters are sampled from posteriors displayed in Supplemental Fig. 2A. Specifically, parameters values are λ_1_ = 0.17 day^−1^, λ_2_ = 1.51 × 10^−3^ day^−1^, c = 0.063 mg/(day·mm^3p^·kg), α = 3.86 × 10^−3^ mg/(mm^3p^·kg), d = 0.29 day^−1^· mm^−2^, Bk_12_ = 0.068 day^−1^, Bk_21_ = 0.18 day^−1^, Bk_e_ = 0.78 day^−1^, Fk_12_ = 0.04 day^−1^, Fk_21_ = 0.88 day^−1^, Fk_e_ = 0.83 day^−1^, K_F_ = 0.94 day^−1^· mm^−2^, α_2_ = 188.37 mg/nl, p_1_ = 19.56. (**A**) Shows how the experimentally observed mean tumour volume varies over a time period of 45 days where the bars represent the standard error of the mean (**B**) shows how the corresponding best fit model solutions varies over the same time period (solid lines) as well as predicted model solutions for combination treatments (dashed lines). (**C**) Shows boxplots corresponding to tumour volumes recorded at 45 days. The coloured dashed line corresponds to the mean and the solid black line corresponds to the median. (**D**) Upper plot shows the bvz and FOLFOX drug concentrations in the plasma for TS1 as predicted by the model, and the lower plot shows the predicted bvz and FOLFOX drug concentrations in the plasma for Treatment Schedule 2 as predicted by the model. Solutions to the ODE system are saved only at times corresponding to experimental measurements. Initial conditions for tumour volume were chosen to be the first non-zero value from experimental data and initial conditions for the carrying capacity was taken to be this value multiplied by 5. The total normalised root mean squared error for the vehicle data, bvz data and FOLFOX data is 0.38.

Figure 4(A) shows the average tumour volume (with standard error) as observed within HCT116 CRC xenograft TS1 studies. These data suggested that there was reduced benefit in administering bvz 24 hrs before FOLFOX. In fact the effects of FOLFOX appeared nullified by administering bvz first. This was consistent with the findings from our mathematical model (see previous section) and may be due to vasculature disruption which hampers FOLFOX penetration of the tumour. Unlike in the mathematical model results presented in the previous section, it was not possible to control the precise initial tumour volume experimentally when testing different drug treatments. This led to some variation in the average tumour volume evolution prior to treatment administration. However, this problem can be overcome by setting the initial tumour volume in the mathematical model equal to the first non-zero recorded experimental value which allowed us to compare the modelling simulations with the data in a direct way.

We performed an extensive fitting (see Methods for details) of the mathematical model to vehicle, bvz and FOLFOX xenograft data and then, using the parameter set which yielded the least squares error, we simulated the effect of combination therapy (TS1) whereby chemotherapy is given 24hrs after bvz and Treatment Schedule 2 (TS2) whereby chemotherapy is given 24hrs before bvz and displayed these results in Fig. 4(B). In Fig. 4(C) we also show a more detailed view of the HCT116 CRC xenograft tumour volume at 45 days (n = 6 mice remain at this time point). All treatments showed a reduction in both the mean and median tumour volumes although none of these are significant. We also show the predicted drug curves for TS1 and TS2 in Fig. 4(D) from the mathematical model. These curves were closely related to the experimental setup because the timing of treatments and dosages were taken directly from the corresponding experiments. In addition, we also simultaneously performed a model selection (see methods section for details) and found the mathematical model with the threshold chemotherapy response to be the most probable model of the data. This implies that the vasculature has a switch-like relationship with FOLFOX delivery. In other words, the model predicted that if the vasculature density is reduced sufficiently by bvz delivery, FOLFOX delivery becomes negligible.

In Supplemental Fig. 2(A) we show the final posterior distributions produced by the approximate Bayesian computation algorithm. These distributions [displayed on the diagonal of Supplemental Fig. 2(A)] indicate the most likely value of parameters for reproducing the data with broader distributions indicating less sensitive parameters and narrower distributions indicating more sensitive parameters. For example, the parameter BK_21_, the transfer rate of bvz from the peripheral compartment to central compartment, was particularly robust to change while the tumour growth rate, λ_1_, was particularly well constrained. We have also included the relative sensitivities as computed by inverting the covariance matrix of the final probability distribution as in^21^, see Supplemental Fig. 3(B) where it is shown that the growth constant was the most sensitive parameter. We can also derive relationships between the model parameters and these are displayed in the off-diagonal positions of Supplemental Fig. 2(A). It can be observed that a strong positive relationship existed between the vasculature recruitment rate, c, and d, the rate of endogenous inhibition of tumour vasculature. This was consistent with intuition because if the recruitment rate is smaller, then the inhibition rate will have to be smaller to compensate in order to reflect the data (and vice versa). A less intuitive relationship that was uncovered was the strong negative relationship between the bvz elimination rate, Bk_e_, and the stimulator clearance rate, α. This relationship exists because if bvz is eliminated more rapidly, then more vasculature is required to deliver more bvz and this requires a reduced stimulator clearance rate.

Finally, in addition to reproducing the monotherapy cases and predicting the combination treatment cases the parameterised model can also be used to predict various further outcomes such as what would happen if treatment was stopped for a break of three weeks following combination treatments before resuming treatment (we show this case in Supplemental Fig. 4(A)) or how different dosages of bvz and FOLFOX impact the tumour reduction (see heatmaps in Supplemental Fig. 5). The model can also be used to predict responses if treatment commenced earlier or if other treatment strategies, such as administering two doses of FOLFOX followed by bvz or using different delays between treatments, as was studied in^22^.

### The mathematical model captures the qualitative and quantitative experimental data for monotherapies and predicts the outcome of delivering FOLFOX before bvz

**Figure 5:**
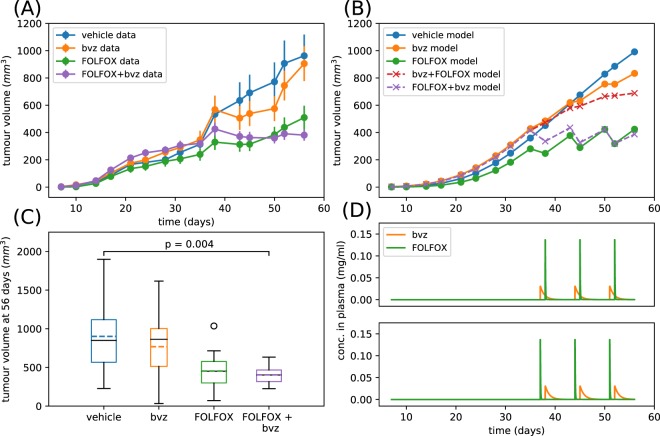
Fitting and validating the computational vascular tumour model with Treatment Schedule 2 (TS2) experimental data. Result of fitting the extended Argyri *et al*. model to experimental data from TS2 HCT-116 xenograft study. Bayesian model selection was used and the threshold model was found to be the most probable given the data. Parameters are sampled from posteriors displayed in Supplemental Fig. 3. Specifically, parameters values are λ_1_ = 0.085 day^−1^, λ_2_ = 2.51 × 10^−3^ day^−1^, c = 0.60 mg/(day·mm^3p^·kg), α = 5.59 × 10^−3^ mg/(mm^3p^·kg), d = 0.80 day^−1^ mm^−2^, Bk_12_ = 0.45 day^−1^, Bk_21_ = 0.008 day^−1^, Bk_e_ = 0.78 day^−1^, Fk_12_ = 0.05 day^−1^, Fk_21_ = 0.22 day^−1^, Fk_e_ = 0.50 day^−1^, K_F_ = 1.65 day^−1^ mm^−2^, α_2_ = 720.8 mg/nl, p_1_ = 12.24. (**A**) Shows how the experimentally observed tumour volume varies over a time period of 56 days where the bars represent the standard error of the mean (**B**) shows how the corresponding best fit model solutions varies over the same time period (solid lines) as well as predicted model solutions for combination treatments (dashed lines). (**C**) Shows boxplots corresponding to tumour volumes recorded at 56 days. The coloured dashed line corresponds to the mean and the solid black line corresponds to the median. (**D**) Upper plot shows the bvz and FOLFOX drug concentrations in the plasma for Treatment Schedule 1 as predicted by the model and the lower plot shows the predicted bvz and FOLFOX drug concentrations in the plasma for TS2 as predicted by the model. Initial conditions for tumour volume were chosen to be the first non-zero value from experimental data and initial conditions for the carrying capacity was taken to be this value multiplied by 5. The total normalised root mean squared error for the vehicle data, bvz data and FOLFOX data is 0.24.

Figure 5(A) shows the average tumour volume with standard error computed from HCT116 CRC xenograft monotherapy and TS2 studies. As in Fig. 4(A), we can see that variation in the initial tumour volumes led to differences in the temporal evolution of the average tumours pre-treatment. In this case, this was exaggerated as treatment was not able to begin until day 37 and only 3 rounds of treatment were able to be performed before animals were euthanized. This was overcome in the model by again setting the tumour initial conditions and drug administration times appropriately.

As for the TS1 case, for the TS2 case we performed an extensive fitting of the mathematical model to the vehicle data, bvz data and FOLFOX data and then using the parameter set which yielded the least squares error we simulated the effect of combination therapy (TS1 and TS2) (Fig. 5(B)). In Fig. 5(C) we also show a more detailed view of the HCT116 CRC xenograft tumour volume at 56 days (n = 12 mice remain at this time point). As for TS1, all treatments showed a reduction in both the mean and median tumour volumes but this time we observed a statistically significant decrease in the tumour volume for TS2 (FOLFOX + bvz). We also show the predicted curves for TS1 and TS2 in Fig. 5(D). Furthermore, while fitting the model we simultaneously performed a model selection and found again the mathematical model with the thresholded chemotherapy response to be the most probable model of the data. This reinforces the hypothesis that the tumour vasculature dependent FOLFOX delivery displayed a threshold or in other words a switch-like response to bvz.

In Supplemental Fig. 2(B) we show the final posterior distributions produced by the approximate Bayesian computation algorithm. The relationships between parameters appeared unchanged compared to the TS1 fitting presented in the previous section, but some parameters have shifted, for example, the tumour growth rate appeared to be much larger. To investigate these differences in more detail, we also computed the distances (specifically the Kolmogorov-Smirnov statistic) between the final parameter distributions shown in Supplemental Fig. 2(A,B) and show these in Supplemental Fig. 3(A). This quantified which model parameters have changed the most between the two different experiments. As expected, the tumour growth rate, λ_1,_ changed the most (this can be seen by simply comparing the tumour volume at the same time between the two different experiments). The computed distance also showed less obvious changes between experiments, such as the vasculature recruitment rate, c, and the half maximal concentration of vasculature for FOLFOX mediated degradation, α_2_. Thus the mathematical model allowed us to gain insights into the underlying biology across different experiments. As in the previous section, we also show the parameter sensitivities in Supplemental Fig. 3(C) and note that this parameterised model can now be used to make a number of additional predictions. To demonstrate this, we show the predicted tumour evolution following a three week break with no treatment before resuming treatment for another three weeks (shown in supplemental Fig. 4(B)). We note that in this case the long term evolution of the tumour with just FOLFOX treatment is very similar to the evolution of the tumour with FOLFOX + bvz (though the FOLFOX + bvz combination is still superior in reducing the tumour volume). This suggests that the FOLFOX was more potent in reducing tumour volume than bvz for this experiment. Finally, we also show the predicted tumour reduction for different doses of bvz and FOLFOX (displayed in Fig. 5(B)).

### Functional interrogation of treatment sequencing effects reveals a complex time dependent response

**Figure 6:**
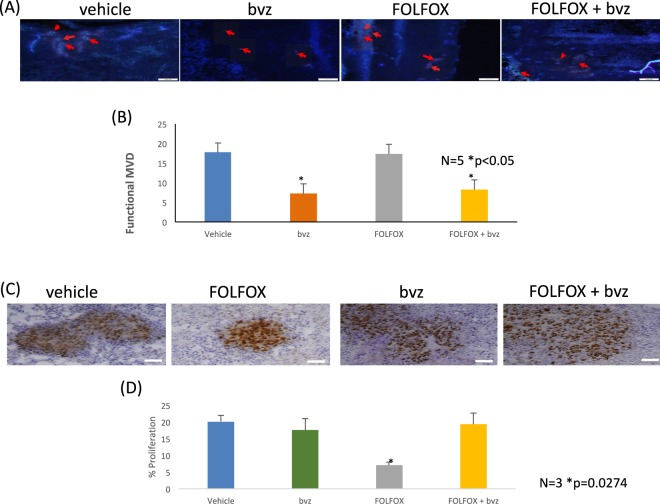
Four weeks of FOLFOX + bvz combination therapy regardless of treatment sequence does not significantly affect proliferation, necrosis or microvessel density in CRC xenograft. (**A**) Representative micrographs of HCT116 tumours treated with FOLFOX and bvz combination therapy for 4 weeks, analysed for % necrosis, proliferation (Ki67) and microvessel density (CD31). In H&E images areas outlined by dotted lines are areas of necrosis. Ki67 positive cells are stained brown while total nuclei are stained blue. Arrows point to positive vessels in CD31 images. Image analysis data for (**B**) % necrosis, (**C**) proliferation index and (**D**) microvessel density. Neither MVD nor % necrosis were significantly different between treatment group, or between treatment schedules after 4 weeks of FOLFOX and bvz combination therapy. Bvz treated tumours in both treatment schedules display a significant (p = 0.0474 TS1 and p = 0.024 TS2) decrease in proliferation by 4 weeks. Error bars represent SEM. N = 3 for all experiments.

While the mathematical model can provide insights into the macroscale evolution of the tumour, it does not provide detailed information on the tumour specific molecular effects of combinatorial treatment. As such, further biological assays were warranted. At the end of 4 weeks, animals were euthanized and tumours were excised and probed using markers of proliferation (Ki67), micro-vessel density (MVD) (CD31/PECAM1) and cell death (necrosis via H&E staining). Neither cell proliferation, death nor microvessel density were significantly different in tumours which underwent either TS1 or TS2 (Fig. 6(A,B)). However bvz monotherapy treated tumours in T1 displayed a small but significant increase (Fig. 6B) in proliferation after 4 weeks compared to vehicle treated tumours whereas bvz monotherapy treated tumours in T2 displayed a small but significant decrease (TS1 p = 0.0474, TS2 p = 0.024 N = 3 per group).

**Figure 7:**
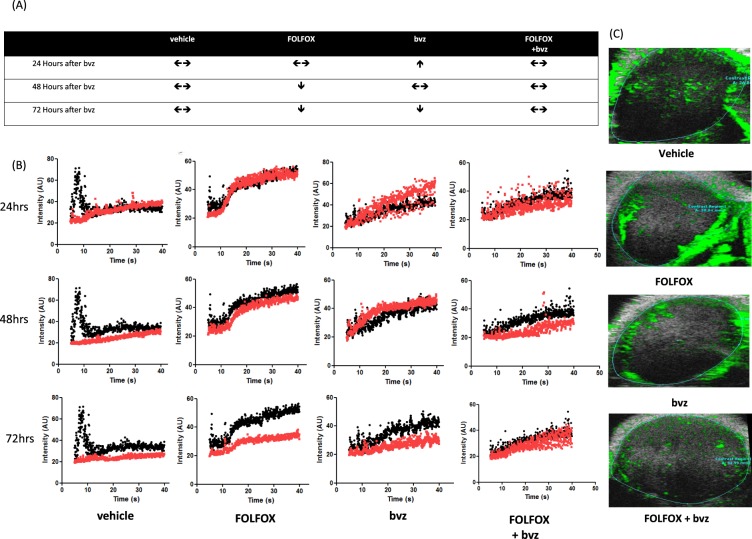
CEUS analysis displays maintenance of blood flow in FOLFOX + bvz treated tumours at early time points. Baseline 2D kinetic CEUS was performed on HCT116-Luc2 xenograft tumours 3 days before commencement of treatment. CEUS was again performed 24 hrs after bvz (as per schedule TS2) and then again 48 and 72 hrs later. (**A**) Table describing average blood flow dynamics compared to baseline flow in each treatment group.  is an increase in blood flow,   is maintenance of flow and  is a decrease in flow. N = 5 per group. (**B**) Representative kinetic wash in curve (time *vs*. intensity) analysis of tumor blood flow for all treatments and time points. Black line represents baseline blood flow. Red line represents blood flow at time point indicated (**C**) Representative contrast enhanced ultrasound image of tumors at 72hrs post treatment. Green areas represent areas of blood flow.

To further explore the efficacy of TS2 (which replicates a common clinical drug scheduling scenario whereby initial dose of bvz is given following chemotherapy) we performed additional early time-point contrast enhanced ultrasound (CEUS) and immunohistochemistry (IHC) studies. 5 animals per cohort were analysed for blood flow kinetics in the first 72 hrs after commencement of therapy. Figure 7(A,B) show average tumour blood flow in TS2 treated tumours at early time points. Tumours treated with FOLFOX alone showed no change in blood flow dynamics within the first 24 hour period in 3 out of 4 tumours (blood flow decreased in 1 out of 4 tumours at this time), but subsequently showed a slow decline in flow over the subsequent 48 hours in all 4 animals. Bvz monotherapy treated tumours showed a marked increase in blood flow in 3 out of 5 tumours with the remainder remaining stable, during the first 24 hr window suggesting normalization of the tumour blood network. However, blood flow subsequently returned to base line with a further decrease observed by 72 hrs (5 out of 5 tumours). Tumours treated with FOLFOX followed by bvz 24hrs later showed no change in blood flow dynamics in 4 out of 5 tumours (1 out of 5 had decreased blood flow) over the 72 hour analysis period. These data suggested a balance between the ostensible inhibitory effect of FOLFOX alone on tumour blood flow vs the early positive effect of bvz on tumour blood flow. Figure 7C shows representative CEUS images with limited blood flow within the tumour core in both FOLFOX monotherapy and bvz monotherapy treated animals after 72 hrs.

**Figure 8:**
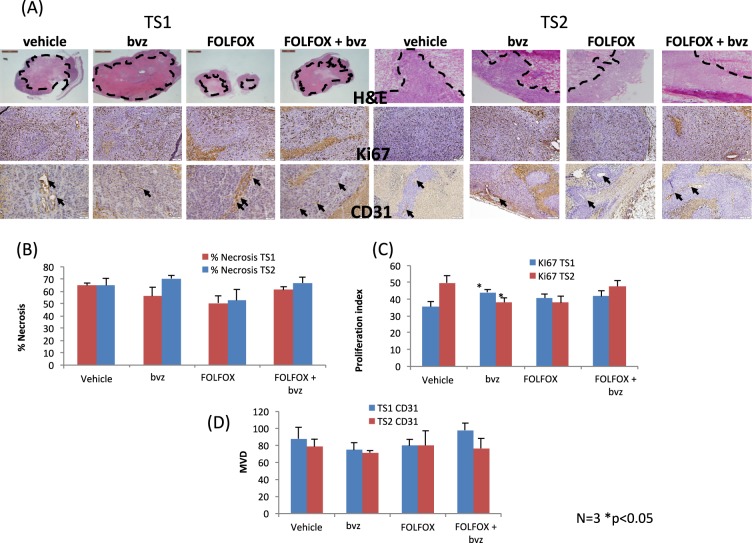
One week of FOLFOX + bvz combination therapy decreases functional vessels in a HCT-116 CRC xenograft model. (**A**) Representative images of the tumours stained with H33342. Red arrows indicate stained vessels within the tumour mass. (**B**) Image analysis of the tumours stained with H33342. As vessels only with blood flowing through them are stained we measure the functional microvessel density (fMVD) of the tumour sections. Mice treated with bvz or FOLFOX followed by bvz show a significant (p = 0.01455 BVZ, P = 0.02432 FOLFOX and bvz) decrease in the number of functional vessels after one week. White bar represents 100 µm. Error bars are SEM. N = 5. (**C**) Representative images of tumours probed with α-Ki67, a proliferation marker. Cells that are actively proliferating stain brown with DAB. Total cell nuclei are stained with haemotoxilin in blue. (**D**) Image analysis of α-Ki67. Proliferation index of HCT116 tumours after one week of treatment. FOLFOX treated tumour display a significant (p = 0.0274) decrease in Ki67 staining after one week of therapy. Error bars represent SEM N = 3.

Following CEUS, mice were injected intravenously with H33342 fluorescent dye and humanely euthanized after 1 minute. This dye which circulates in the blood, stains nuclei of blood vessel endothelial cells, thus providing information about functional vasculature (fMVD). Figure 8(A) shows fMVD of tumours treated with vehicle, FOLFOX, bvz or FOLFOX followed by bvz. Tumours treated with either bvz or with FOLFOX + bvz showed a striking decrease in the fMVD (p = 0.01455 and p = 0.02432 respectively) while vehicle and FOLFOX treated tumours showed no change in the number of functional vessels. A significant decrease (p = 0.0274) in proliferation (Fig. 8(B)) was observed in tumours treated with FOLFOX after 72hrs.

## Discussion

The main objective of this study was to investigate anti-angiogenic drug scheduling in the context of CRC. This was achieved by combining clinically relevant xenograft studies and computational modelling. Both approaches suggest that administering FOLFOX first may be optimal in this disease setting, with CRC xenograft data demonstrating that scheduling FOLFOX prior to bvz yields a 60.4% average reduction in tumour size compared with 36.3% when bvz is delivered prior to FOLFOX (p < 0.05). The computational model was further calibrated to xenograft data which was then used to make additional predictions regarding optimal combinations of drugs and how the tumour would evolve if treatment was interrupted for a break.

In this study we extended an ODE model describing vascular tumour growth under angiogenic signalling presented in^18^ to account for FOLFOX treatment. To this end, the pharmacokinetic properties of FOLFOX have been incorporated into the model and the combination therapy effect has been simulated for FOLFOX and bvz. One potential weakness of the 2016 study, alluded to by the authors, was the fact that the parameter fitting was performed only locally and different parameter sets were used to fit to the control and bvz treatment cases. Hence, we also extended the study to perform a comprehensive global fitting of the model using Bayesian parameter fitting techniques to two novel experimental data sets (producing normalised root squared mean errors of 0.38 and 0.24). This was made possible due to the economic computational cost of running our model, though we note that there have been recent advances in the simulation of more holistic cellular automaton models^23^. It may even be possible to calibrate such models with spatio-temporal data in the near future though it will require careful acquisition of three dimensional *in vivo* imaging data for effective calibration as discussed in^24^.

Across several major cancer indications, the most effective application of anti-VEGF therapy relies upon combination with cytotoxic drugs. Nevertheless, there is an ongoing debate about the underlying mechanisms involved. As mentioned, one long-held view is that anti-angiogenic therapies enhance efficacy of cytotoxic drugs by “normalizing” structurally and functionally abnormal tumor vessels, thereby reducing interstitial fluid pressure and improving drug penetration^7^. Nevertheless, several studies have also shown that bvz leads to a sustained decrease in the delivery of biological agents or chemotherapy^9–11^. Overall it seems likely that the tumour vessel effect of anti-angiogenics is likely to be a complex time, dose, tumour and even tumour vessel phenotype dependent^11–13,25,26^ phenomenon. Moreover, while the study of vascular tumour growth using mathematical models has been the subject of several papers^27^, the study of combinatorial anti-angiogenic regimens in this context, has received less attention. Combinatorial treatment regimens were applied to a simple cellular automaton model of glioblastoma in^28^ and the model was shown to reproduce qualitative aspects of pre-clinical and clinical data. However, only treatment parameters were studied, and other parameters were taken from previous studies. In our study we have inferred all parameters from experimental data. In^15^, it was suggested that the chemotherapy uptake could be enhanced by first delivering anti-vascular treatment to prune leaky vessels, ensuring maximal drug flow to the tumour. This was corroborated by a recent study in breast cancer which suggested that bvz should be delivered 2.2 days before chemotherapy in order to optimize tumour burden reduction^22^. Our modeling and experimental xenograft data suggest that the opposite treatment strategy (i.e., delivering chemotherapy first followed by bvz) may be optimal in the context of CRC.

Employing a gold-standard ectopic HCT-116 CRC xenograft model we successfully managed to ‘reverse translate’ a common clinical drug scheduling scenario whereby initial dose of bvz is delivered following chemotherapy (TS2). We have further employed this model to compare outcome when treatment sequence is reversed and bvz is delivered prior to chemotherapy (Figs 6 and 7). Notwithstanding inherent limitations of subcutaneous cell line xenograft models, we have nevertheless recapitulated mCRC clinical response to combinatorial treatment when FOLFOX is delivered prior to anti-VEGF showing a significantly increased anti-tumour response for the TS2 sequence. Thus, our computational predictions and experimental data suggest that FOLFOX delivered before bvz (TS2) may be most advantageous in the CRC setting.

As the enhanced TS2 response could not be explained at study termination (4 weeks of treatment) by analyses of fMVD, tumour cell proliferation or necrosis, we thus performed additional functional imaging (CEUS) and IHC analyses to further interrogate TS2 response mechanisms and establish early time point effects on blood flow, functional vessel and tumour cell proliferation. Initial early vessel normalization (24 h) with bvz monotherapy was as expected but we observed decreased flow over time (48–72 h) and reduced number of functional vessels at 1 week (vessel pruning). This ‘vascular normalization window’ and multi-modal normalization/subsequent pruning effect of anti-VEGF therapy which is time and dose dependent has previously been proposed (e.g.^29^). While there was no overall effect of FOLFOX monotherapy on the number of functional vessels after 1 week, we nevertheless observed a decrease in blood flow over early time-points with FOLFOX monotherapy (48–72 h). Both anti- and pro-angiogenic effects of 5-FU based chemotherapy have variously been observed across different tumour types^30,31^ and are therefore likely to be tumour and organ site dependent. However, when combined, FOLFOX + bvz appears to preserve blood flow at early time-points (24–72 hr) not withstanding an ostensible decrease in the number of functional vessels (vessel pruning) at week 1 as seen in the bvz monotherapy cohort. Further studies are required to fully understand the impact of chemotherapy and bvz on vessel function specifically within the context of CRC. Longitudinal functional and investigational imaging studies (e.g. positron emission tomography (PET), functional magnetic resonance imaging (MRI), multispectral fluorescence ultra-microscopy) in metastatic patients are specifically warranted.

The ODE model developed can now be implemented as a predictive tool for clinically relevant rodent studies, employing orthotopic and patient derived xenograft (PDX) models which better represent inter and intra-tumoural heterogeneity and molecular subtypes^32^. It is also possible that the response to different treatment sequences might further depend on CRC consensus molecular subtypes (CMS) having different stromal phenotypes^33^. This aspect could also be evaluated in PDX models representing CMS1–4 tumour subtypes. Ultimately, model predictions will require validation in relevant clinical studies such as the ongoing OBELICs trial, a Phase 3 study which seeks to optimize bvz scheduling in combination with chemotherapy in mCRC patients^34^.

In conclusion, our study demonstrates that mathematical models will continue to play an increasingly important role in unravelling and predicting complex combinatorial treatment scheduling effects across tumour types and specific drug combinations.

## Methods

### Computational Model

We used an ordinary differential equation model to capture the tumour volume changing in time in response to different drug treatment regimes. We adopted this approach because the data we have is primarily in the form of tumour volume time series and hence by saving the output from our ODE model at times corresponding to the experimentally measured tumour volumes, we could make a fair comparison between the model and data. The ODE model we used to simulate vascular tumour growth is defined in great detail in^18^ but we summarise here the key assumptions.

The mathematical model is governed by a pair of ordinary differential equations which reflect the interplay between tumour volume (*V*) and the vasculature or carrying capacity (*K*). The primary assumptions on which the model is built are the following. We assumed the tumour has spherical symmetry and that the diffusion process via which pro and anti-angiogenic factors are transported is in a quasi-stationary state. We also assumed that the concentration of the stimulator is a radially symmetric, continuously differentiable function and that the clearance rate of proangiogenic factors is a monotonically increasing function of drug concentration and it is always greater than the respective value in the absence of treatment. Finally, we assumed the change of drug concentration inside the tumour caused by the dysfunctional vasculature is governed by the proportionality to a bounded and decreasing function of tumour volume.

The initial basis of the model was developed by Hahnfeldt *et al*.^20^ and was refined in^19^, leading to the following two ODE system

1 $$\frac{dV}{dt}=-\,{\lambda }_{1}Vln(\frac{V}{K})$$

2 $$\frac{dK}{dt}=-\,{\lambda }_{2}K+c(\frac{\beta {V}^{p}V}{\alpha (\beta +{V}^{p})+{I}_{1}(t)})-{\rm{d}}K{V}^{2/3}$$

where *V* stands for tumour volume, *K* for carrying capacity, *I*_*1*_(*t*) for bvz concentration and λ_1_, λ_2_, *c*, *d*, *β*, *α* and *p*, for parameters explained in Table 1. The terms in equation 1 reflect the Gompertzian growth of the tumour which depends on a time-dependent carrying capacity. The first term in equation 2 reflects the intrinsic loss rate which was assumed to be proportional to the carrying capacity. The second and third terms, reflecting stimulatory and inhibitory action induced by tumour cells respectively, were computed by applying a diffusion-consumption equation for the concentration of stimulators and inhibitors. The second term also reflects the suppressive effect of antiangiogenic treatment when bvz treatment is simulated. All coefficients are non-negative, except for the proportionality constant *c* where only positive values are allowed in order to preclude the biologically irrelevant behaviour of an untreated tumour with self-regressing carrying capacity.

**Table 1:** Description of the variables and parameters used in the computational vascular tumour growth and pharmacokinetic models.

Mathematical Symbol	Description	Units	Uniform Prior Range	Reference
t	Time	day	n/a	—
V	Tumour volume	mm^3^	n/a	—
K	Tumour carrying capacity	mm^3^	n/a	—
λ_1_	Gompertziangrowth constant	day^−1^	[0.05, 0.5]	^18– 20^
λ_2_	Degradation rate related to endothelial cells half life	day^−1^	[0, 0.01]	^18– 20^
c	Vasculature recruitment rate by tumour	mg/(day·mm^3p^·kg)	[0, 10]	^18– 20^
d	Endogenous inhibition of tumour vasculature	day^−1^· mm^−2^	[10^−5^, 10^0^]	^18, 20^
α	Stimulator clearance rate	mg/(mm^3p^·kg)	[0, 10]	^18, 19^
β	Extent of the abnormal phenotype of tumour vasculature	mm^3p^	1	^19^
p	Extent of the abnormal phenotype of tumour vasculature	—	0	^19^
I_1_	Concentration of bvz in plasma	mg/ml	—	—
I_2_	Concentration of FOLFOX in plasma	mg/ml	—	—
D_1_	Dosage of bvz	mg	10	Experiment
D_2_	Dosage of FOLFOX	mg	56	Experiment
T	Infusion duration	day	1/48	Experiment
V_c_	Volume of central compartment	ml	7.975	^18, 35^
w	Average weight of mouse	kg	0.025	Experiment
K_F_	Tumour cell degradation rate due to FOLFOX	day^−1^ mm^−2^	[0, 1]	^40^
Bk_12_	Transfer rate of bvz from central to peripheral compartment	day^−1^	[0, 1]	^18, 35^
Bk_21_	Transfer rate of bvz from peripheral to central compartment	day^−1^	[0, 1]	^18, 35^
Bk_e_	Rate of bvz elimination	day^−1^	[0, 1]	^18, 35^
Fk_12_	Transfer rate of FOLFOX from central to peripheral compartment	day^−1^	[0, 1]	^18, 35^
Fk_21_	Transfer rate of FOLFOX from peripheral to central compartment	day^−1^	[0, 1]	^18, 35^
Fk_e_	Rate of FOLFOX elimination	day^−1^	[0, 1]	^18, 35^
α_1_	Extent of tumour volume dependence on FOLFOX-mediated tumour degradation	—	[−3, 3]	—
_β1_	Extent of vasculature dependence on FOLFOX-mediated tumour degradation	—	[−3, 3]	—
p_1_	Hill coefficient of vasculature for FOLFOX mediated degradation	—	[0, 20]	—
α_2_	Half-maximal concentration of vasculature for FOLFOX mediated degradation	mg/ml	[0, 1000]	—

In 2016, Argyri *et al*., a further refinement of this model was made to give the correct asymptotic behaviour in the limit of large doses of bvz. In this limit, the tumour returns to the prevascular volume (i.e., the tumour volume prior to the angiogenic switch) which we denote by V^*^, this leads to the system of equations

3 $$\frac{dV}{dt}=-\,{\lambda }_{1}(V-{V}^{\ast })ln(\frac{V-{V}^{\ast }}{K-{V}^{\ast }})$$

4 $$\frac{dK}{dt}=-\,{\lambda }_{2}(K-{V}^{\ast })+c(\frac{\beta ({(V-{V}^{\ast })}^{p})(V-{V}^{\ast })}{\alpha (\beta +{(V-{V}^{\ast })}^{p})+{I}_{1}(t)})-{\rm{d}}(K-{V}^{\ast }){(V-{V}^{\ast })}^{2/3}$$

Note that the terms in equations 3 and 4 are the same as in equations 1 and 2 except that they have the prevascular volume subtracted from each terms. We began from this system of equations and extended it to consider the impact of FOLFOX treatment

5 $$\frac{dV}{dt}=-\,{\lambda }_{1}(V-{V}^{\ast })ln(\frac{V-{V}^{\ast }}{K-{V}^{\ast }})-{{\rm{K}}}_{F}{I}_{2}(t)f(V-{V}^{\ast },K-{V}^{\ast })$$

6 $$\frac{dK}{dt}=-\,{\lambda }_{2}(K-{V}^{\ast })+c(\frac{\beta ({(V-{V}^{\ast })}^{p})(V-{V}^{\ast })}{\alpha (\beta +{(V-{V}^{\ast })}^{p})+{I}_{1}(t)})-{\rm{d}}(K-{V}^{\ast }){(V-{V}^{\ast })}^{\frac{2}{3}}$$

While bvz directly impacts the vascular compartment, we made the additional assumption that FOLFOX directly impacts the tumour volume compartment – this is reflected in the second term of equation 5.

The pharmacokinetic properties of bvz and FOLFOX were modelled using a two compartment model with first order elimination which has been shown to be appropriate in previous studies^35–37^. We also made the assumption that there is no direct interaction between FOLFOX and bvz as was reported in^38^. Taking into account that the treatments are administered via the intravenous infusion, a zero order absorption assumption was made which reflects steady drug delivery into the patient’s or mouse’s systemic circulation. The central compartment represents blood and highly perfused tissues such as lungs, liver and kidneys and the volume of which is represented by the parameter V_c_. The elimination of drugs is assumed to occur from the central compartment according to the first order transfer rate FKe for FOLFOX or BKe for bvz. This was assumed to be where most elimination takes place due to the function of the liver and kidneys. The outer compartment was taken to correspond to poorly perfused tissue. Since monitoring of the drug concentration at the anatomical region of interest is not feasible, the concentration of drug in plasma was assumed to reflect the concentration in the site of the target tissue. Hence, the equations characterising the central compartment are the ones which impact the computational vascular tumour growth model. We assumed the duration of infusion is the same for both drugs and we represent the duration time by parameter T. The dosages of bvz and FOLFOX are represented by D_1_ and D_2_ respectively. The time point of drug administration is represented by t_D_ and varies depending on the treatment. While the infusion is taking place, i.e., t − t_D_ ≤ T:

$${I}_{i}(t)=\frac{{D}_{i}}{T}[\frac{{A}_{i}}{{a}_{i}}(1-{e}^{-{a}_{i}(t-{t}_{D})})+\frac{{B}_{i}}{{b}_{i}}(1-{e}^{-{b}_{i}(t-{t}_{D})})],{\rm{for}}\,{\rm{i}}=1,2,$$

whereas in the post-infusion period, i.e., T < t − t_D:_

$${I}_{i}(t)=\frac{{D}_{i}}{T}[\frac{{A}_{i}}{{a}_{i}}(1-{e}^{-{a}_{i}T}){e}^{-{a}_{i}(t-{t}_{D}-T)}+\frac{{B}_{i}}{{b}_{i}}(1-{e}^{-{b}_{i}T}){e}^{-{b}_{i}(t-{t}_{D}-T)}],\,{\rm{for}}\,{\rm{i}}=1,2,$$

Where i = 1 represents bvz and i = 2 represents FOLFOX and the remaining variables are defined as

$${a}_{1}=\frac{B{K}_{21}B{K}_{e}}{{b}_{1}},$$

$${b}_{1}=0.5(B{K}_{12}+B{K}_{21}+B{K}_{e}-\sqrt{{(B{K}_{12}+B{K}_{21}+B{K}_{e})}^{2}-4B{K}_{21}B{K}_{e}}),$$

$${A}_{1}=\frac{1}{{V}_{C}}\frac{{a}_{1}-B{K}_{21}}{{a}_{1}-{b}_{1}},{B}_{1}=\frac{1}{{V}_{C}}\frac{{b}_{1}-B{K}_{21}}{{b}_{1}-{a}_{1}},$$

and

$${a}_{2}=\frac{F{K}_{21}F{K}_{e}}{{b}_{2}},$$

$${b}_{2}=0.5(F{K}_{12}+F{K}_{21}+F{K}_{e}-\sqrt{{(F{K}_{12}+F{K}_{21}+F{K}_{e})}^{2}-4F{K}_{21}F{K}_{e}}),$$

$${A}_{2}=\frac{1}{{V}_{C}}\frac{{a}_{2}-F{K}_{21}}{{a}_{2}-{b}_{2}},{B}_{2}=\frac{1}{{V}_{C}}\frac{{b}_{2}-F{K}_{21}}{{b}_{2}-{a}_{2}},$$

Further details on the pharmacokinetic properties of the two compartment model can be found in^39^ and parameters involved in the pharmacokinetic model are explained in Table 1.

We considered two different functional forms of f – the function which dictates the manner in which FOLFOX eradicates the tumour volume. The first way we modelled this is using the common form outlined in^40^, i.e.,

7 $$f(V-{V}^{\ast },K-{V}^{\ast })={(V-{V}^{\ast })}^{{\alpha }_{1}}{(K-{V}^{\ast })}^{{\beta }_{1}}$$

which is a second order reaction or bimolecular reaction form used in many mathematical biology applications. It can be interpreted as the rate of tumour degradation being proportional to the tumour volume and the vasculature compartment size. The exponents α_1_ and β_1_ provide further flexibility and allow the model to capture nonlinear dependencies of FOLFOX treatment on tumour volume or the carrying capacity. We hereby refer to it as the “Continuous model”. The second form we considered is given by the formula

8 $$f(V-{V}^{\ast },K-{V}^{\ast })=(V-{V}^{\ast })\frac{{(K-{V}^{\ast })}^{{p}_{2}}}{{{\alpha }_{2}}^{{p}_{2}}+{(K-{V}^{\ast })}^{{p}_{2}}}$$

Here, the rate of tumour degradation is linearly proportional to the volume of the tumour and nonlinearly proportional to the vasculature compartment in a saturating manner. In the limit of large p_2_, this sigmoidal function becomes switch-like. In other words, if the vasculature abundance is very low and underdeveloped there will be negligible delivery of FOLFOX while if the vasculature is very large in abundance there will be an upper limit on the amount of FOLFOX that can be delivered. We hereby refer to this model as the “Threshold model”.

### Numerical simulation and parameter estimation

All numerical simulations were performed in Julia using the DifferentialEquations.jl package^41^ using the CVode backward differentiation formula solver^42^. It is worth noting that the numerical experiments concerning free tumour growth as well as tumour growth under intermittent drug treatments have been performed on a desktop computer with an Intel Xeon 2.5Ghz processor, 32GB RAM in Windows 7 with 64-bit operating system. Using this system, we used the BenchmarkTools.jl package in Julia to find the following benchmark statistics for the model. The average running time for sampling the model from the prior parameter distributions defined in Table 1 was 7.45 milliseconds and the median time was 5.68 milliseconds (based on 670 sample simulations).

We approached this problem with the aim of obtaining biologically plausible and testable predictions. Hence, rather than inferring a narrow range of parameter values, we aimed to analyse a wide range of the parameter space. The short run time of our chosen model framework allowed us to explore the parameter space thoroughly using Approximate Bayesian Computation (ABC) methods. In particular, we employed the ABC method outlined in^43^. This method allowed us to fit the model to the data, perform parameter sensitivity analysis and Bayesian model selection. In brief, ABC-SMC is a particle filter approach that starts by first generating N particles from the prior. We chose N equal to 10,000 throughout the paper. In the following iterations, the algorithm selects a particle from the obtained distribution, perturbs it, and then simulates the model using that parameter combination. If the obtained model data is sufficiently similar to the observed data that we are trying to fit, the parameter combination is accepted. As soon as N parameter combinations are accepted, these replace the prior, and the algorithm starts anew. As the number of iterations increases, the acceptance threshold approaches zero, such that the simulated data needs to be more and more similar to the observed data. As a summary statistic, we used a squared Euclidean distance metric on the tumour volume time series data (shown in Figs 4A and 5A) for the vehicle and two monotherapy data sets. I.e., we aimed to find the parameter set which fits all three sets of data simultaneously. With respect to model selection, we used the same approach as in^43^ in which a “model parameter” m ∈ {1, 2} was introduced as an additional discrete parameter. We then defined model-specific parameters as θ(1) = (λ_1_, λ_2_, c, α, d, Bk_12_, Bk_21_, Bk_e_, Fk_12_, Fk_21_, Fk_e_, K_F_, α_1_, β_1_) and θ(2) = (λ_1_, λ_2_, c, α, d, Bk_12_, Bk_21_, Bk_e_, Fk_12_, Fk_21_, Fk_e_, K_F_, α_2_, p_1_) for the continuous and threshold model respectively. To begin the model selection we sample a model indicator m from the prior distribution π(m) which we chose to be a discrete uniform prior distribution (i.e. both models are assumed to be equally likely at the beginning). For model m we then propose new particles by perturbing the particles from the previous population specific to m; this step is the same as in the parameter estimation algorithm. The weights for particles θ(m) are also calculated like in the parameter estimation algorithm for m. Hence parameter inference and model selection are done simultaneously.

### Tumour cell culture

HCT-116-luc CRC cells were obtained from Caliper Life Sciences. Cells were grown in RPMI 1640 medium supplemented with 10% FBS, 2mM L-Glutamine, 100 U/mL penicillin and 100 μg/mL streptomycin. Cells were cultured at 37 °C in 5% CO_2_ and maintained in logarithmic growth by sub- culturing a minimum twice weekly.

### CRC Xenograft Model

Animal experiments conformed to guidelines from Directive 2010/63/EU of the European Parliament on the protection of animals used for scientific purposes. Experiments were licensed and approved by the Department of Health and Children Dublin Ireland project license number B100/3654. Ethics protocols were also reviewed by University College Dublin Animal Research Ethics Committee under protocol number P12–27. 48 Balb C^nu/nu^ mice (Charles River Laboratories, UK) were subcutaneously implanted with 2 × 10^6^ HCT116 cells in the right flank as previously described^44^ and tumours were allowed to grow until they reached 200 mm^3^. Subsequently, animals were divided into cohorts (n = 12) and treated with either vehicle (5% glucose and PBS) or the previously determined clinically relevant doses of bvz [10 mg/kg, IP once a week] and FOLFOX [**Fol**inic acid 13.4 mg/kg, 5-**F**U: 40 mg/kg, **Ox**aliplatin: 2.4 mg/kg., IP once a week 24hrs after bvz] either alone or in combination for 4 weeks (see Supplemental Fig. 1(A,B) for treatment schedules). FOLFOX constituents for rodent studies were calculated according to^45,46^. 40 mg/kg 5-FU bolus equates to a plasma concentration of 35.8 mg/L while 2400 mg/m^2^ infusion for standard human (modified de Gramont standard protocol^47^) gives plasma concentration of 27.4 mg/L min – 43.2 mg/L max over the infusion. Oxaliplatin and Folinic acid do not interfere with the absorption or clearance 5-FU. Therefore dose of oxaliplatin and folinic acid were accordingly titred to mouse and in line with 5-FU dosing.

Tumour response was assessed by calliper measurement. At 4 weeks all animals were euthanized and tissue was collected for further immunohistochemical (IHC) analyses. All animal experiments were performed by authorized researchers in accordance with animal research guidelines (Health Products and Regulatory Authority, Ireland). Protocols were further approved by the Animal Research Ethics Committee (AREC) at University College Dublin. Animals were housed in groups, maintained on a 12 hr light/dark cycle, with access to food and water ad libitum.

### Immunohistochemistry

#### End of study analyses

Paraffin-embedded, formalin-fixed tissue sections from HCT116-luc2 ectopic tumours taken after 4 weeks of both TS1 and TS2 therapy were stained with Hematoxylin and Eosin (H & E) in order to access percentage of tumour core necrosis. Anti-KI67 and anti-PECAM1/CD31 staining procedures were carried out to determine tumour cell proliferation and microvessel density respectively. In brief, tumour sections were deparaffinized with xylene and rehydrated according to standard histologic procedures. For epitope retrieval, sections were boiled for 20 min in 10 mM citrate buffer, pH 6.0. Sections were rinsed in phosphate buffered saline (PBS)-Tween 20 (0.1% v/v). Subsequently slides were processed according Lab vision Ultravision polymer detection system (Thermoscientific, UK) in conjunction with the Lab Vision Autostainer 360 following manufactures instructions. Sections were probed with rabbit anti-Ki-67 (Millipore, Billerica, MA), dilution 1:150;and rabbit anti-CD31 (Santa Cruz Biotechnology, Inc., Dallas, TX), dilution 1:75 in PBS for 1 hr. Positive signal was developed with diaminobenzidine (Life Sciences UK,) according to the manufacturer’s instructions for 10 mins Sections were counterstained with haematoxylin to visualize nuclei. For assessment of proliferation indices (percentage of KI-67- positive cells), fractions of labelled tumour cells were assessed in 10 microscopic high-power fields per section using x40 magnification and ImageJ software (National Institutes of Health, Bethesda, MD). Microvessel density based on criteria previously described (Leonard *et al*., 2008) was assessed.

#### Early time-point analyses

Functional microvessel density (fMVD): Frozen H33342 labelled tumours n = 5 taken after one week of TS2 therapy were sectioned (8 µm thick) on a cryostat at −20 C. Sections were allowed to dry in the open air and were then visualized using a Zeiss Axiovert fluorescent microscope with a 384 nm filter. fMVD was determined as previously described (Leonard *et al*., 2008). Briefly CD31 images were analyzed by applying a 15000 pixel^2^ grid over the image in Image J and counting the number of times positive vessels cross the grid.

Proliferation and Necrosis: Frozen tumours n = 3 taken after one week of TS2 therapy were sectioned (8 µm thick) on a cryostat at −20 C. Sections were then fixed in 10% formalin for 15 mins and washed with PBS. Sections were stained with Hematoxylin and Eosin (H&E) to assess percentage necrosis. Anti- Ki67 staining was carried out to determine tumour cell proliferation. Sections were probed with rabbit anti-Ki-67 (Millipore, Billerica, MA), dilution 1:150 using the Labvision Ultravision (Thermo scientific) polymer detection system.

### Contrast Enhanced Ultrasound (CEUS)

20 Balb C^nu/nu^ mice (Charles River Laboratories, UK) were implanted with 2 × 10^6^ HCT116 cells in the right flank (as described above) and tumours allowed to grow until they reached 200 mm^3^. 72hrs before treatment (TS2), high-frequency CEUS was performed on a dedicated small-animal, high-resolution imaging system (Vevo 770 VisualSonics, Amsterdam) with a 40-MHz transducer; All mice (*n* = 20) were thus imaged at baseline in 2D contrast mode.

Specifically, prior to the imaging session, mice were anesthetized with 2.0% isoflurane with 0.8 L/min oxygen and placed on a computer controlled heated platform set at 37 C. Warmed ultrasound gel was placed on the ectopic tumour. The probe (RMV 704B; VisualSonics, Inc.) was positioned so that the transducer imaged the centre of the tumour mass. The probe was then adjusted until the tumour was visible on the screen of the ultrasound unit within the focal zone. The parameters of the ultrasound system were set at 100% transmitted power, 10.00 × 10.00-mm field of view. To obtain contrast-enhanced images, a 50-μL bolus of contrast agent (MicroMarker; VisualSonics) containing approximately 1 × 10^8^ microbubbles, was injected into the lateral tail vein via catheter and mechanical pump at a rate of 0.6 mL/min for 5 seconds. Imaging commenced 5 seconds prior to contrast injection to generate a “no contrast” baseline. The recording continued for 800 frames.

Animals were subsequently grouped into 4 cohorts of n = 5 and treated according to TS2 as described above. 24, 48 and 72 hrs after bvz animals underwent further 2D CE-US scans (Supplemental Fig. 1(C)) After 7 days of treatment animals were anaesthetized as described above. Subsequently, H33342 dye (40 mg /kg in PBS) was injected in the lateral tail vein with. The dye was allowed to circulate for 1 minute to fluorescently label all vessels. Animals were subsequently euthanized and tumours flash frozen in liquid nitrogen and stored at −80.

## Electronic supplementary material

Supplementary figures
