# Author Correction: Anti-angiogenic drug scheduling optimisation with application to colorectal cancer

**DOI:** 10.1038/s41598-018-31456-9

**Published:** 2018-09-27

**Authors:** M. Sturrock, I. S. Miller, G. Kang, N. Hannis Arba’ie, A. C. O’Farrell, A. Barat, G. Marston, P. L. Coletta, A. T. Byrne, J. H. Prehn

**Affiliations:** 1 Department of Physiology and Medical Physics, Centre for Systems Medicine, Royal College of Surgeons in Ireland, 123 St Stephens Green, Dublin, 2 Ireland; 2 School of Medicine, University of Leeds Brenner Building, St James’s University Hospital, Leeds, LS9 7TF UK; 3 Conway Institute, University College Dublin, Belfield, Dublin 4 Ireland; 4 Liverpool Hope University, Hope Park, Liverpool, L16 9JD UK

Correction to: *Scientific Reports* 10.1038/s41598-018-29318-5, published online 25 July 2018

In the original version of this Article, A. T. Byrne and J. H. Prehn were omitted as jointly supervising authors. This has now been corrected in the PDF and HTML versions of the paper. The Supplementary file was correct at the time of publication.

