# Supplementary material for: Anti-angiogenic drug scheduling optimisation with application to colorectal cancer
Source: Sci Rep. 2018 Jul 25;8:11182. doi: 10.1038/s41598-018-29318-5 (PMC6060139; doi:10.1038/s41598-018-29318-5)
Supplement: Supplementary file 1 — Supplementary figures [file 41598_2018_29318_MOESM1_ESM.pdf]

Supplemental Figures for ``**Anti-angiogenic drug scheduling optimisation with application to colorectal cancer**''

Sturrock M[1]*, Miller IS[1]*, Kang G[1], Hannis Arba'ie N[1], O'Farrell AC[1], Barat A[1], Marston G[4], Coletta PL[2], Byrne AT[1,3#], Prehn JH[1#].

[1] Department of Physiology and Medical Physics, Centres for Systems Medicine, Royal College of Surgeons in Ireland, 123 St Stephens Green, Dublin 2, Ireland

[2] School of Medicine, University of Leeds Brenner Building, St James's University Hospital, Leeds LS9 7TF, UK

[3] Conway Institute, University College Dublin, Ireland

[4] Liverpool Hope University, Hope Park, Liverpool, L16 9JD

* Equal Contribution

# Joint Corresponding Authors

Experimental work flow

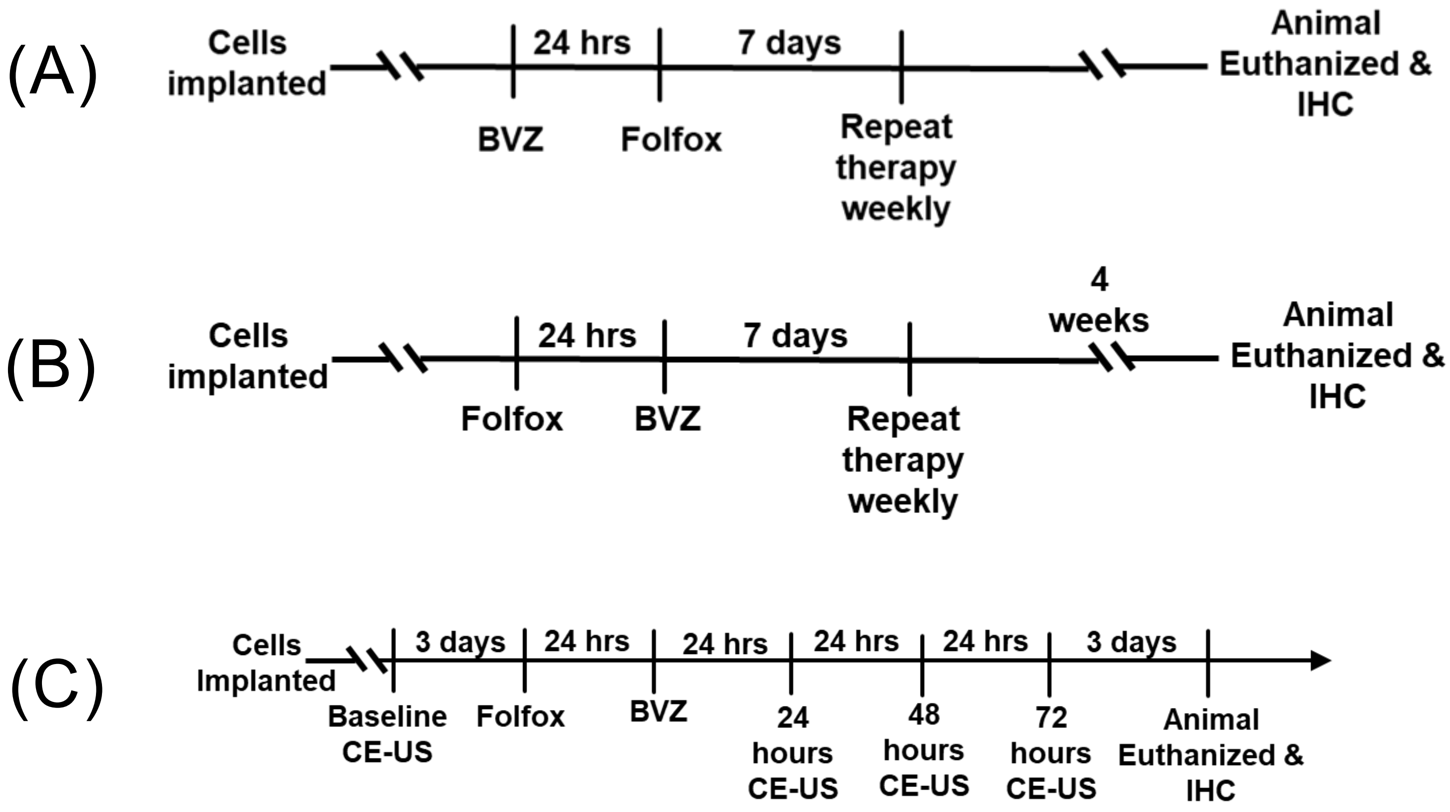

(A)

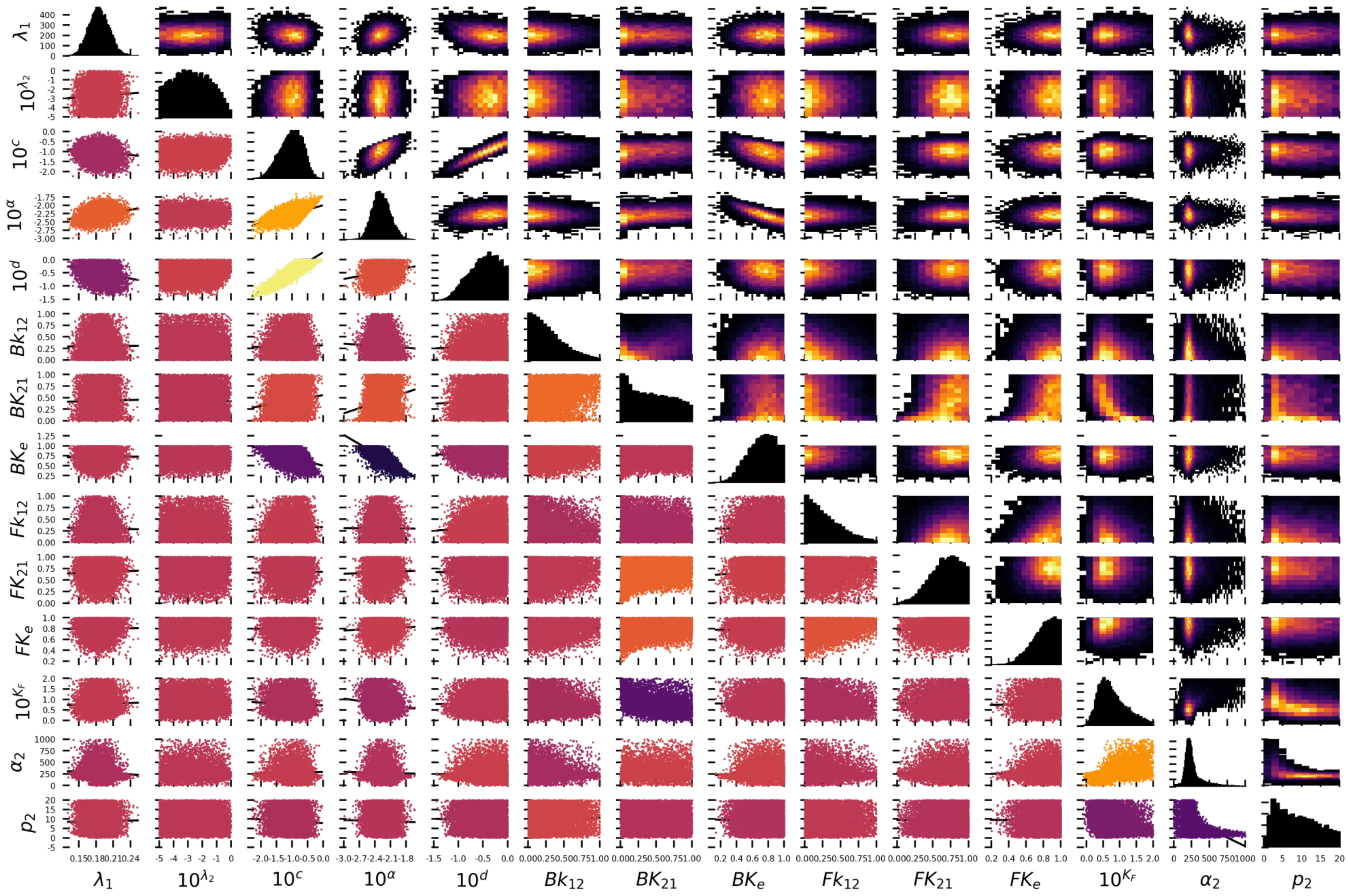

λ_1
10^{λ_2}
10^c
10^α
10^d
Bk_{12}
BK_{21}
BK_e
Fk_{12}
FK_{21}
FK_e
10^{K_F}
α_2
p_2

(B)

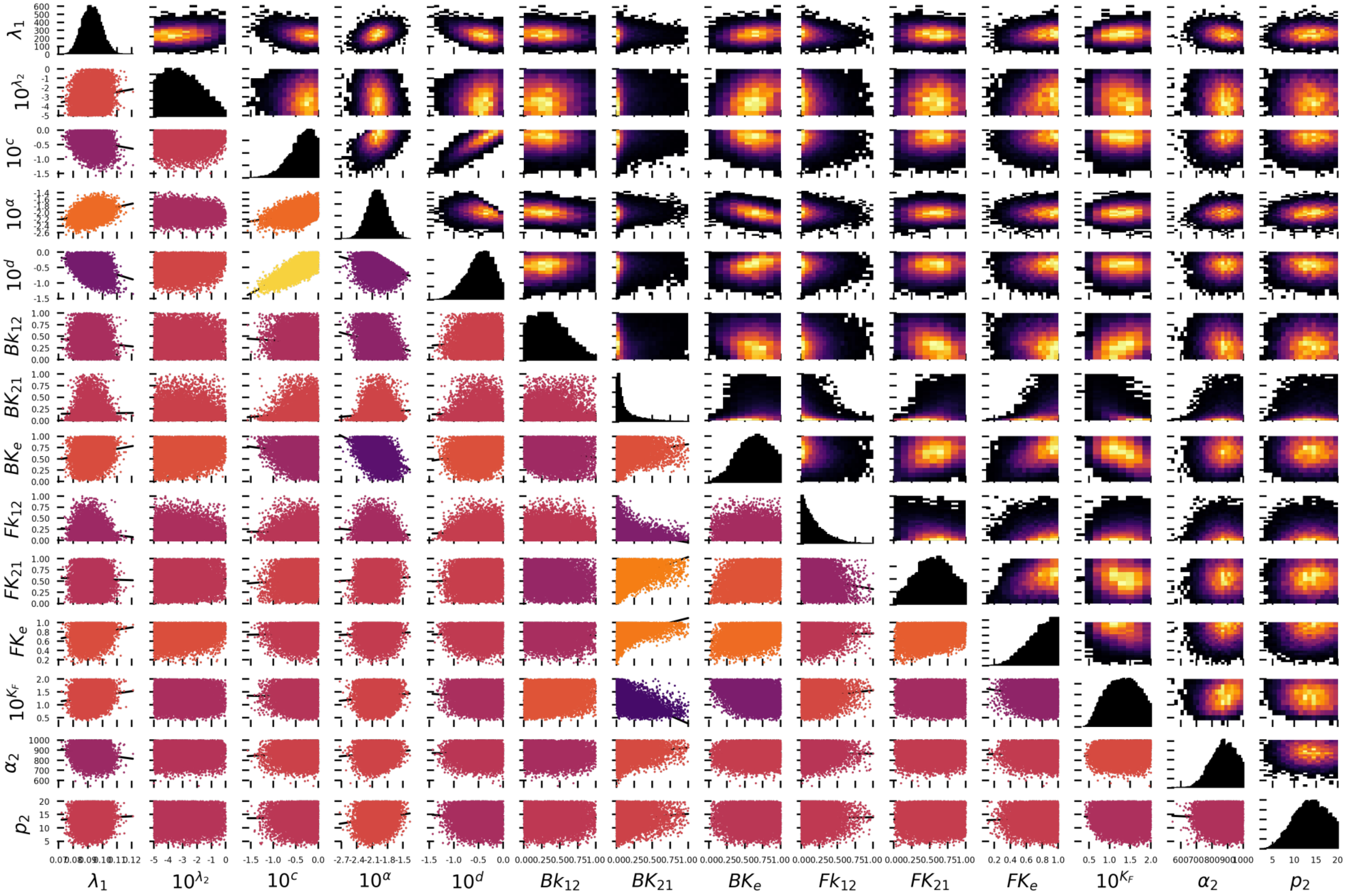

λ_1
10^{λ_2}
10^c
10^α
10^d
Bk_{12}
BK_{21}
BK_e
Fk_{12}
FK_{21}
FK_e
10^{K_F}
α_2
p_2

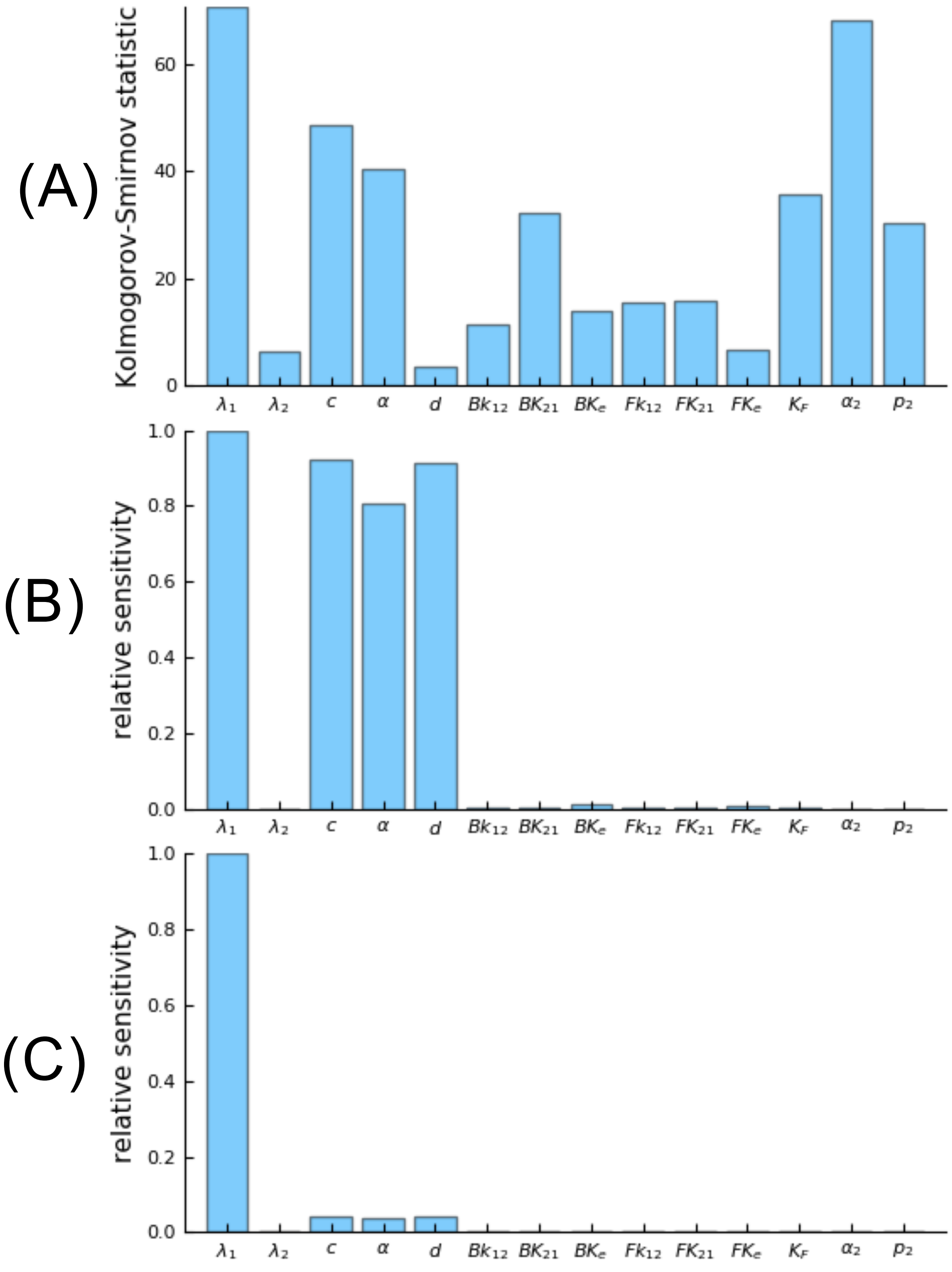
(A)
Kolmogorov-Smirnov statistic
0
20
40
60
λ1 λ2 c α d Bk12 BK21 BKe Fk12 FK21 FKe KF α2 p2
(B)
relative sensitivity
0.0
0.2
0.4
0.6
0.8
1.0
λ1 λ2 c α d Bk12 BK21 BKe Fk12 FK21 FKe KF α2 p2
(C)
relative sensitivity
0.0
0.2
0.4
0.6
0.8
1.0
λ1 λ2 c α d Bk12 BK21 BKe Fk12 FK21 FKe KF α2 p2

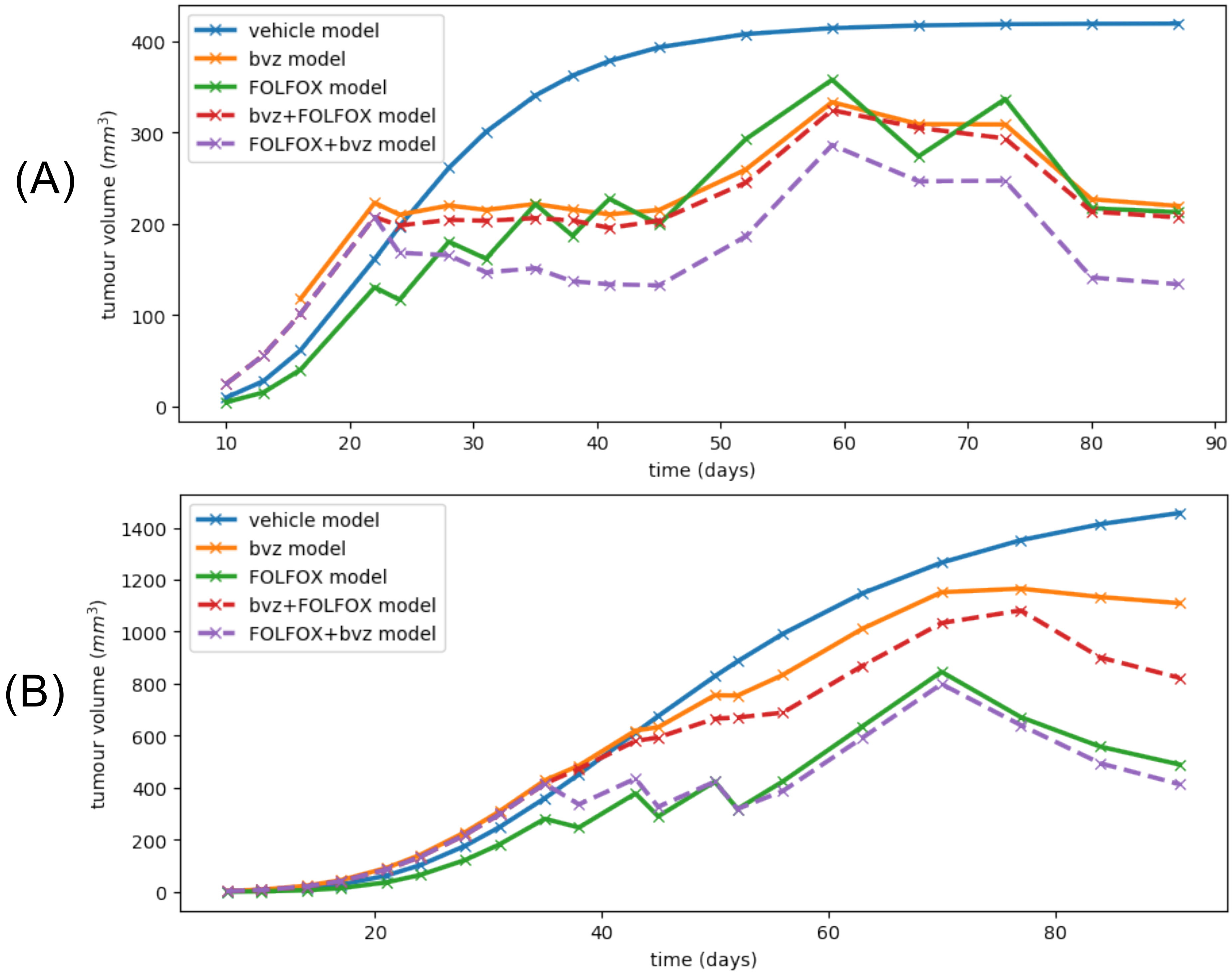
(A)
vehicle model
bvz model
FOLFOX model
bvz+FOLFOX model
FOLFOX+bvz model
tumour volume (mm^3)
time (days)
0
100
200
300
400
10
20
30
40
50
60
70
80
90
(B)
vehicle model
bvz model
FOLFOX model
bvz+FOLFOX model
FOLFOX+bvz model
tumour volume (mm^3)
time (days)
0
200
400
600
800
1000
1200
1400
20
40
60
80

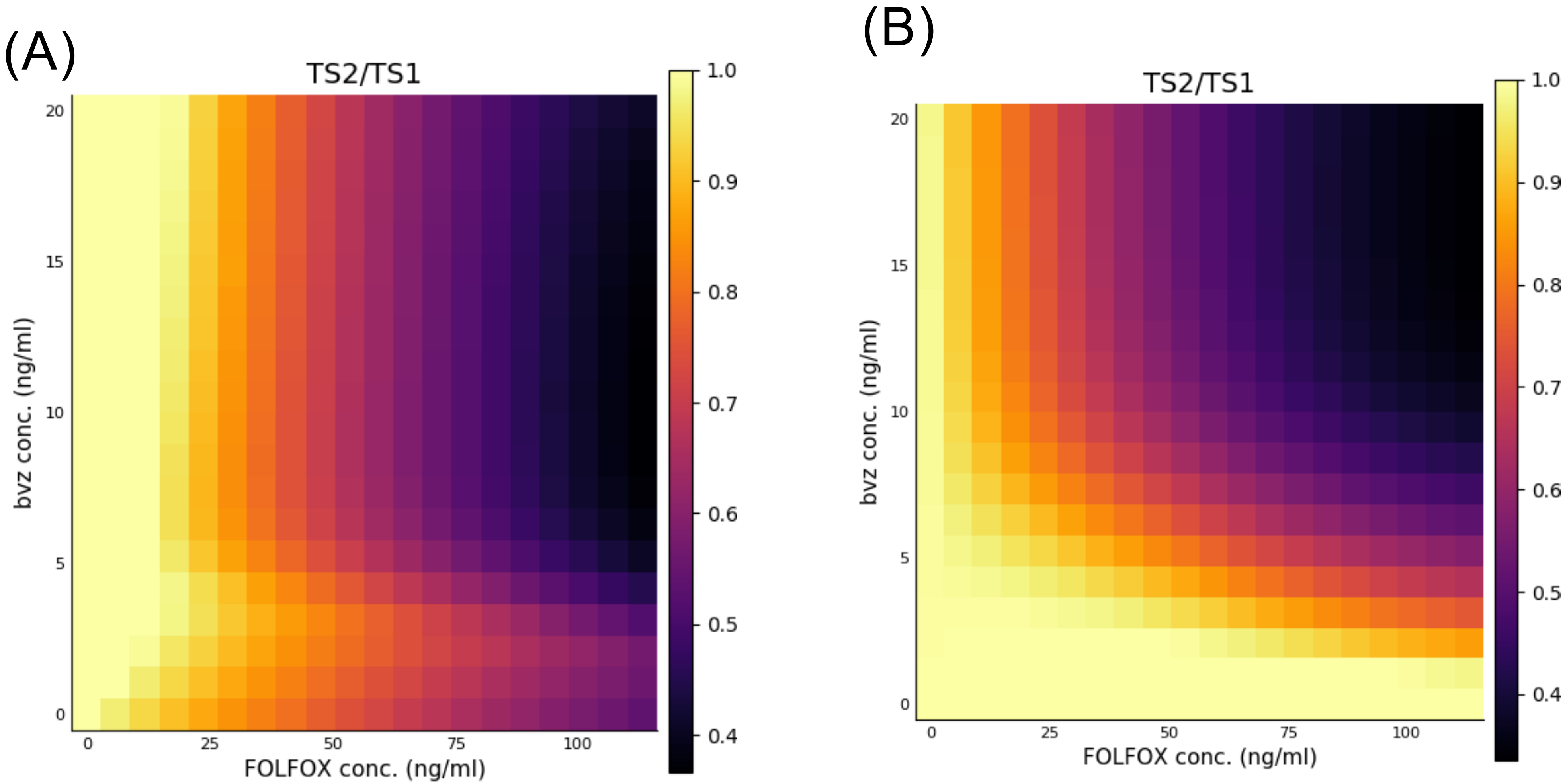

(A)
TS2/TS1
bvz conc. (ng/ml)
FOLFOX conc. (ng/ml)
0
25
50
75
100
5
10
15
20
0.4
0.5
0.6
0.7
0.8
0.9
1.0
(B)
TS2/TS1
bvz conc. (ng/ml)
FOLFOX conc. (ng/ml)
